# Response to an *Escherichia coli* K88 oral challenge and productivity of weanling pigs receiving a dietary nucleotides supplement

**DOI:** 10.1186/s40104-015-0049-5

**Published:** 2015-12-02

**Authors:** Hanlin Li, Pinyao Zhao, Yan Lei, Tianshui Li, InHo Kim

**Affiliations:** Department of Animal Resource & Science, Dankook University, No. 29 Anseodong, Cheonan, Choongnam, 330-714 South Korea

**Keywords:** Dietary nucleotides, *Escherichia coli* K88 challenge, Growth performance, Immunity, Nutrient digestibility, Weanling pigs

## Abstract

**Background:**

Dietary nucleotides, considered as antibiotics alternative, were shown to have positive effects on intestinal hyperaemia, systemic immunity, small-intestinal growth, and hepatic composition in pigs. However, there is no previous research on nucleotide supplementation in weanling pigs under an oral challenged *E. coli* K88. Therefore, 2 experiments were conducted to investigate the effects of dietary nucleotides on weanling pig growth performance, nutrient digestibility, fecal score, and blood profile after being orally challenged with *E. coli* K88.

**Methods:**

In Exp. 1, a total of 140 weanling pigs [8.33 ± 0.33 kg of body weight (BW), 28-d old] were used in this 42-d feeding trial. Pigs were distributed into 1 of 4 treatments, 5 pigs/pen (3 barrows and 2 gilts) and 7 pens/treatment. Treatments were a control basal diet (CON) or the CON supplemented with 150 (R150), 220 (R220), or 275 (R275) mg/kg to give the three treatment diets. In Exp. 2, 28 weanling pigs (BW = 8.40 ± 0.22 kg, 28-d old) were distributed into 1 of 4 treatments to give 1 pig/pen and 7 pens/treatment in a 42-d feeding and challenge trial. Dietary treatments were the same as in Exp. 1. On d 14, all those pigs (BW = 13.3 ± 0.15 kg, 42-d old) were orally dosed with 1.5 mL suspension containing 10^10^ cfu/mL of *E. coli* K88. Twenty four hours after challenge, blood and excreta samples were collected from each pigs for analysis. Fecal scores were measured on d 7, 14, 21, and 28 of the study.

**Results:**

In Exp. 1, overall BW, average daily gain (ADG), gain/feed (G/F) ratio, and nutrient digestibilities were lower (*P* < 0.05) in CON group compared with the nucleotides fed pigs. In Exp. 2, after challenge, IgA, IgM, and IGF-I were higher (*P* < 0.05) in the nucleotide groups compared with CON. However, the nucleotide groups had lower (*P* < 0.05) cortisol and TNF-α compared with CON. Fecal *E. coli* counts and fecal score for the nucleotide groups were lower (*P* < 0.05) than for CON.

**Conclusions:**

In conclusion, dietary nucleotides supplementation could improve growth performance, nutrient digestibility, immune status, microbial balance, reduce diarrhea, and provide protection against enterotoxigenic *E. coli* K88 infection in weanling pigs.

## Background

Previously, antibiotics were used in feed to overcome weaning-associated problems in the swine industry. Due to the risk of antibiotic resistance in humans, many countries have already banned the use of antibiotics in livestock feeds from 2006. Therefore, it is important to find alternatives to antibiotics [1]. Various substitutes for antibiotics, such as probiotics, prebiotics, oligosaccharides, enzymatic preparations, plant extract, and Chinese medicinal herbs have been assessed [2]. Dietary nucleotides, which are also considered as one of these alternatives, were shown to have positive effects on intestinal hyperaemia [3], immunity stimulation [4], small-intestinal growth [5], and hepatic composition [6] in pigs and rodents.

Weanling is accompanied with morphological, histological, and microbial changes in the gastrointestinal tract of young mammals and yet the amount of *de novo* synthesis of nucleotides is insufficient to meet gut requirements [7]. Furthermore, it has been shown that nucleotides accounts for as much as 20 % of the non-protein fraction of milk in most mammalian specie [8]. Also, nucleotides are naturally present in all foods of animal and plant origin [9], and as bioactive agents they may have potential to reduce challenges related to weaning [10]. To this end, dietary nucleotides supplementation has been shown to prevent pig diarrhea [11].

Furthermore, dietary nucleotides supplementation may be beneficial during periods of rapid growth and development, disease challenges, injury, and stress, because they play an important role in enhancing immunity, maintaining intestinal health, and preserving energy. However, studies on nucleotide supplementation in challenged weanling pigs are limited. Thus, the objectives of the present study were to evaluate the effects of dietary nucleotides supplementation on growth performance, nutrient digestibility, fecal consistency score, and blood immune responses in weanling pigs and to determine whether dietary nucleotides supplementation could improve immunity of weanling pigs orally challenged with *E. coli* K88.

## Methods

The experimental protocols describing the management and care of animals were reviewed and approved by the Animal Care and Use Committee of Dankook University. The nucleotide (Rovimax™ NX; DSM Nutritional Products Ltd, Basel, Switzerland) product used in the present study is a hydrolyzed dehydrated yeast extract biomass (*Kluyveromyces Fragilis*) enriched with free nucleotides. To indicate the dietary nucleotides content, nitrogen of the sample was measured and crude protein (CP) was calculated by using CP = nitrogen (N) × 6.25 (CP ≥ 75 %, loss on drying < 8 %, ashes ≤ 30 %). According to a previous study [12], Rovimax™ NX was designed to provide the same amount of nucleotides per d as sow’s milk by including in post-weaning diets.

### Exp.1: Feeding trial

A total of 140 weanling pigs [(Landrace × Yorkshire) × Duroc, 28 ± 1 d-old, n = 35] with an average BW of 7.25 ± 0.33 kg (no creep feed was offered during lactation) were used in this 42-d experiment. Pigs were randomly allotted to 1 of 4 dietary treatments according to their initial BW and sex to give 7 replicate pens per treatment with 5 pigs (3 barrows and 2 gilts) per pen (2.0 m × 2.0 m). Dietary treatments were: CON, basal diet; R150, CON + 150 mg/kg nucleotide; R220, CON + 220 mg/kg nucleotide; R275, CON + 275 mg/kg nucleotide. As shown in Table 1, diets used in the present study were formulated to meet or little exceed the estimated nutrient requirements for weanling pigs recommended by NRC [13]. All pigs were housed in an environmentally regulated room with slatted plastic flooring and mechanical ventilation system, while lighting was automatically regulated to provide 12 h of artificial light per day. Initial temperature in the room was maintained at 30 ± 1 °C and humidity at around 60 %, then temperature decreased by 1 °C each week during the experimental period. Each pen was equipped with an one-sided, stainless steel self-feeder and one nipple drinker which allowed pigs access to feed and water for *ad libitum* consumption throughout the experimental period.

**Table 1:** Composition of basal diets (as-fed basis)^a^

Item	d 1 to 7	d 8 to 21	d 22 to 42
Ingredient, %			
Extruded corn	11.15	34.92	45.10
Extruded oat	10.00	-	-
Bakery by products	-	5.00	9.00
Soybean meal (44 % CP)	8.00	20.00	29.65
Fermented soybean meal	7.80	8.20	-
Fish meal	5.00	4.00	2.50
Soy oil	4.15	4.80	3.00
Lactose	10.00	6.00	-
Whey	17.00	10.70	6.85
Milk product	13.00	2.00	2.00
Mono-calcium-phosphate	1.25	1.00	0.60
Sugar	4.00	2.00	-
Plasma powder	6.50	-	-
L-Lys⋅HCl (78 %)	0.12	0.25	0.16
DL-Met (50 %)	0.26	0.15	0.14
L-Thr (89 %)	0.77	0.08	
Choline chloride (25 %)	0.20	0.10	0.10
Vitamin premix^b^	0.10	0.10	0.10
Mineral premix^c^	0.20	0.20	0.20
Limestone	0.20	0.20	0.30
Salt	0.30	0.30	0.30
Calculated composition, %			
ME, kcal/kg	3,540	3,540	3,490
CP	22.00	21.00	20.50
Lys	1.57	1.41	1.33
Met	0.60	0.49	0.47
Ca	0.80	0.78	0.75
Total P	0.76	0.76	0.64
Analyzed composition, %			
ME, kcal/kg	3,546	3,544	3,474
CP	22.80	21.20	20.60
Lys	1.54	1.42	1.37
Met	0.57	0.48	0.46
Ca	0.81	0.75	0.71
Total P	0.74	0.71	0.62

^a^We add the feed composition, which is not including antibiotics, or enzymes

^b^Provided per kilogram of complete diet: vitamin A, 11,025 IU; vitamin D3, 1,103 IU; vitamin E, 44 IU; vitamin K, 4.4 mg; riboflavin, 8.3 mg; niacin, 50 mg; thiamine, 4 mg; d-pantothenic acid, 29 mg; choline, 166 mg; and vitamin B12, 33 μg

^c^Provided per kilogram of complete diet: Fe (as FeSO_4_⋅7H_2_O), 80 mg; Cu (as CuSO_4_⋅5H_2_O), 12 mg; Zn (as ZnSO_4_), 85 mg; Mn (as MnO_2_), 8 mg; I (as KI), 0.28 mg; and Se (as Na_2_SeO_3_⋅5H_2_O), 0.15 mg

Individual BW and pen feed consumption were measured on day 1, 7, 21, and 42 to calculate ADG, average daily feed intake (ADFI), and G/F ratio. Apparent total tract digestibility (ATTD) of dry matter (DM), gross energy (GE), and N were determined using chromic oxide (0.2 %) as an indigestible marker that was added to the diets 7 d before taking samples (during d 1–7, 14–21 and 35–42). Fresh fecal samples were collected from 2 pigs per pen by rectal massage on d 7, 21 and 42 and stored at −20 °C until analyzed. Before chemical analysis, fecal samples were thawed, dried at 60 °C for 72 h and then finely ground to pass through a 1-mm screen. Feed and fecal samples were analyzed for DM and GE in according to the procedures outlined by AOAC [14]. Chromium was analyzed via UV absorption spectrophotometry (Shimadzu, UV-1201, Shimadzu, Kyoto, Japan).

Two pigs from each pen (n = 14 per treatment) in Exp. 1 were randomly selected for blood sampling via anterior vena cava puncture on d 14 (before challenge), 15 (24 h after challenge), 21, and 42. Blood samples were collected into either 5-mL vacuum tubes without and with K_3_EDTA coating (Becton Dickinson Vacutainer Systems, Franklin Lakes, NJ). Serum samples were obtained by centrifuging blood at 3,000 × g for 15 min at 4 °C and serum immunoglobulins were determined using ELISA kits. Whole blood samples were analyzed for red blood cell (RBC), white blood cell (WBC) and lymphocyte counts using an automatic blood analyzer (ADVIA 120, Bayer, Tarrytown, NY, USA).

On d 14 (before challenge), d 15 (24 h after challenge) and d 42, fecal samples were collected via rectal palpation from 2 pigs in each pen, then samples pooled and placed on ice before put in freezer (−20 °C). One gram of the composite fecal sample from each pen was diluted with 9 mL of 1 % peptone broth (Becton, Dickinson and Co., Franklin Lakes, NJ) and homogenized. Samples were the serially diluted 10-fold in 1 % peptone solution and then plated onto MacConkey agar plates (Difco Laboratories, Detroit, MI) and Lactobacilli medium III agar plates (Medium 638, DSMZ, Braunschweig, Germany) to isolate *E. coli* and *Lactobacillus*, respectively. The *Lactobacilli* medium III agar plates were incubated for 48 h at 39 °C under anaerobic conditions. The MacConkey agar plates were incubated for 24 h at 37 °C and colonies were counted immediately after removal from incubator. Fecal scores were determined at 0800 and 2000 during d 1 to 7 and on d 21, 28, 35 and 42 using the following fecal scoring system: 1 = hard, dry pellet; 2 = firm, formed stool; 3 = soft, moist stool that retains shape; 4 = soft, unformed stool that assumes shape of container; 5 = watery liquid that can be poured. The fecal score was determined as the average value for all pigs in each pen as determined using this 5-grade score system [15].

### Exp.2: Challenge trial

A total of 28 weanling pigs (BW = 8.40 ± 0.22 kg, 28-d weaned) were distributed into 1 of 4 treatments in a 42-d trial to give 1 pig/pen and 7 pens/treatment. Experimental diets were the same as in Exp. 1 and were offered for *ad libitum* intake throughout the experimental period. On d 14, pigs (BW = 13.3 ± 0.15 kg, 42-d-old) were orally dosed with 1.5 mL suspension containing 10^10^ cfu/mL of *E. coli* K88. The dosage of *E. coli* K88 was based on a previous study [16]. Twenty four hours after challenge, blood and fresh fecal samples were taken for analysis as described in Exp. 1. Fecal scoring was done on d 21, 28, 35, and 42 using the system described in Exp.1. Individual BW and feed intake were determined on d 1, 14, and 42 to calculate ADG, ADFI and G/F ratio. No vaccines or antibiotics were administered to these pigs before or during the study.

### Statistical analysis

All data were subjected to statistical analysis as a randomized complete block design using the PROC MIXED procedures of SAS (SAS Inst. Inc., Cary, NC) using pen as the experimental unit. The model statement for analyses for overall weaning pigs criteria for the post-weaning period, main effects of diet and replication as a covariate. Contrast was used as: 1) CON vs. mean of the nucleotide groups; 2) R150 vs. R275; 3) R220 vs. mean of R150 and R275. Initial BW was used as a covariate for ADFI and ADG. Before carrying out statistical analysis of the microbial counts, logarithmic conversion of the data was performed. Differences were determined using a *P* < 0.05 indicating significance differences, whereas 0.05 < *P* < 0.10 showed statistical trends.

## Results

### Exp. 1: Feeding trial

#### Growth performance (Table 2)

**Table 2:** The effects of dietary nucleotides supplementation on growth performance of weaned pigs (Exp. 1)^a^

Item	CON	R150	R220	R275	SEM^b^	Contrast^c^		
						1	2	3
Body weight, kg								
d 1	8.33	8.33	8.34	8.34	0.04	0.44	0.68	0.16
d 7	11.75	11.96	12.04	12.02	0.11	0.06	0.69	0.75
d 21	16.92	17.22	17.48	17.56	0.23	0.08	0.32	0.76
d 42	24.74	25.26	25.65	25.88	0.34	0.04	0.21	0.85
Phase 1 (d 1 to 7)								
ADG, g	244	259	264	264	8	0.07	0.69	0.79
ADFI, g	300	306	314	310	4	0.05	0.54	0.25
G/F	0.809	0.846	0.840	0.854	0.028	0.26	0.83	0.78
Phase 2 (d 8 to 21)								
ADG, g	370	376	389	396	15	0.34	0.37	0.87
ADFI, g	618	611	603	609	9	0.32	0.86	0.49
G/F	0.601	0.616	0.65	0.655	0.025	0.19	0.28	0.64
Phase 3 (d 22 to 42)								
ADG, g	559	574	584	595	16	0.20	0.39	0.97
ADFI, g	850	850	844	852	3	0.59	0.59	0.06
G/F	0.657	0.676	0.691	0.699	0.020	0.18	0.43	0.87
Overall (d 1 to 42)								
ADG, g	391	403	412	418	8	0.04	0.21	0.85
ADFI, g	590	589	587	590	3	0.73	0.79	0.42
G/F	0.663	0.684	0.703	0.709	0.013	0.03	0.21	0.71

^a^Abbreviation: *CON* basal diet, *R150* basal diet + nucleotides 150 mg/kg, *R220* basal diet + nucleotides 220 mg/kg, *R275* basal diet + nucleotides 275 mg/kg

^b^Standard error mean

^c^Contrast: 1) CON vs. mean of nucleotide groups; 2) R150 vs. R275; 3) R220 vs. mean of R150 and R275

On d 42, pigs fed nucleotides supplemented diets had higher (*P* < 0.05) BW compared with those fed CON diet. During d 1 to 7, ADFI of pigs fed nucleotides supplemented diets was higher (*P* < 0.05) compared with those fed CON diet, whereas no significant differences (*P* > 0.10) in ADG, ADFI or G/F ratio were observed among treatments during d 8 to 21 and d 22 to 42. Overall (d 1 to 42), ADG and G/F ratio of pigs fed nucleotides supplemented diets were higher (*P* < 0.05) compared with CON.

#### Apparent total tract digestibility (ATTD) measurements (Table 3)

**Table 3:** Effect of dietary nucleotide supplementation on nutrient digestibility of weaned pigs (Exp. 1)^a^

Item, %	CON	R150	R220	R275	SEM^b^	Contrast^c^		
						1	2	3
d 7								
Dry matter	82.48	84.07	84.37	86.24	0.79	0.02	0.07	0.43
Nitrogen	81.53	82.35	82.46	82.40	2.68	0.78	0.99	0.98
Energy	83.05	85.55	85.86	86.32	0.83	0.01	0.52	0.94
d 21								
Dry matter	82.60	83.83	85.02	85.05	1.49	0.26	0.57	0.75
Nitrogen	81.56	83.01	82.77	82.82	1.03	0.29	0.90	0.91
Energy	80.91	82.72	84.43	84.43	0.87	0.01	0.18	0.44
d 42								
Dry matter	80.71	83.91	85.60	83.70	0.97	0.01	0.88	0.15
Nitrogen	81.69	84.89	84.85	84.21	1.02	0.02	0.64	0.82
Energy	82.89	834.16	83.76	83.07	0.84	0.44	0.37	0.88

^a^Abbreviation: *CON* basal diet, *R150* basal diet + nucleotides 150 mg/kg, *R220* basal diet + nucleotides 220 mg/kg, *R275* basal diet + nucleotides 275 mg/kg

^b^Standard error mean

^c^Contrast: 1) CON vs. mean of nucleotide groups; 2) R150 vs. R275; 3) R220 vs. mean of R150 and R275

On d 7, ATTD of DM and GE for the nucleotides supplemented diets were higher (*P* < 0.05) than in the CON diet, whereas the R275 group tended to have higher (*P* = 0.07) ATTD of DM compared with the R150 group. On d 21, dietary nucleotides inclusion treatments had higher (*P* < 0.05) ATTD of GE than CON treatment. On d 42, ATTD of DM and N in the CON diet were lower (*P* < 0.05) than in the nucleotides supplemented diets.

#### Blood profiles (Table 4)

**Table 4:** The effects of dietary nucleotide supplementation on blood profiles of weaned pigs (Exp. 1)^a^

Item	CON	R150	R220	R275	SEM^b^	Contrast^c^		
						1	2	3
d 7								
Lymphocyte, %	23.54	25.38	26.81	27.06	1.66	0.15	0.48	0.77
RBC, 10^6^/μL	6.66	6.68	6.55	6.82	0.24	0.94	0.68	0.50
WBC, 10^3^/μL	17.74	19.40	18.60	18.33	1.48	0.55	0.61	0.89
IgG, mg/dL	162.50	173.63	177.63	176.75	5.74	0.05	0.70	0.73
IgA, mg/dL	41.75	43.50	44.88	44.00	0.55	0.001	0.52	0.11
IgM, mg/dL	6.10	7.40	7.60	8.00	0.60	0.04	0.46	0.93
d 21								
Lymphocyte, %	35.53	38.66	38.98	36.98	1.62	0.17	0.47	0.57
RBC, 10^6^/μL	5.82	6.53	5.96	6.26	0.11	0.003	0.10	0.01
WBC, 10^3^/μL	15.45	17.76	18.10	19.19	0.66	0.001	0.14	0.65
IgG, mg/dL	184.38	205.25	240.13	225.50	5.31	<0.0001	0.01	0.001
IgA, mg/dL	42.25	42.75	44.13	43.25	0.65	0.15	0.59	0.17
IgM, mg/dL	6.10	8.00	8.60	8.60	0.50	0.001	0.39	0.62
d 42								
Lymphocyte, %	34.65	40.51	39.88	42.13	3.12	0.10	0.72	0.71
RBC, 10^6^/μL	6.91	6.48	6.53	6.50	0.21	0.11	0.97	0.88
WBC, 10^3^/μL	22.41	22.94	23.64	24.32	1.31	0.43	0.46	0.99
IgG, mg/dL	348.25	367.88	365.50	420.88	28.59	0.28	0.20	0.42
IgA, mg/dL	41.38	42.25	42.38	45.75	1.12	0.12	0.04	0.25
IgM, mg/dL	11.10	13.40	14.00	15.50	1.20	0.03	0.22	0.76

^a^Abbreviation: *CON* basal diet, *R150* basal diet + nucleotides 150 mg/kg, *R220* basal diet + nucleotides 220 mg/kg, *R275* basal diet + nucleotides 275 mg/kg

^b^Standard error mean

^c^Contrast: 1) CON vs. mean of nucleotide groups; 2) R150 vs. R275; 3) R220 vs. mean of R150 and R275

On d 7, IgA and IgG concentration in piglets fed the nucleotides supplemented diets were higher (*P* < 0.05) compared with those fed the CON diet. On d 21, RBC, WBC, IgG, and IgM in piglets fed the nucleotides supplemented diets were higher (*P* < 0.05) compared with those fed the CON diet, moreover, RBC in the R220 group were higher (*P* < 0.05) than the average of R150 and R275 groups. The concentration of IgG in R275 was higher (*P* < 0.05) than that in R150 whereas its level in the R220 group was higher (*P* < 0.05) than the average of R150 and R275 groups. On d 42, pigs fed the R275 diet had higher (*P* < 0.05) IgA concentration than those fed the R150 diet. Pigs fed the nucleotides supplemented diets had higher (*P* <0.05) IgM concentration compared with the CON group.

#### Fecal microflora (Table 5)

**Table 5:** The effects of dietary nucleotide supplementation on fecal microflora of weaned pigs (Exp. 1)^a^

Item, log_10_cfu/g	CON	R150	R220	R275	SEM^b^	Contrast^c^		
						1	2	3
*Lactobacillus*	6.83	7.08	7.24	7.30	0.14	0.02	0.26	0.76
*E. coli*	6.54	6.13	5.83	5.41	0.17	0.001	0.01	0.76

^a^Abbreviation: *CON* basal diet, *R150* basal diet + nucleotides 150 mg/kg, *R220* basal diet + nucleotides 220 mg/kg, *R275* basal diet + nucleotides 275 mg/kg

^b^Standard error mean

^c^Contrast: 1) CON vs. mean of nucleotide groups; 2) R150 vs. R275; 3) R220 vs. mean of R150 and R275

Fecal *Lactobacillus* counts in pigs fed the nucleotides supplemented diets were higher (*P* < 0.05) compared with CON pigs. The fecal *E.coli* counts for pigs fed the nucleotides supplemented diets were lower (*P* < 0.05) compared with those fed the CON diet. Also, R275 pigs had lower (*P* < 0.05) fecal *E.coli* counts compared with R150 pigs.

#### Fecal score (Table 6)

**Table 6:** The effects of dietary nucleotides supplementation on diarrhea score of weaned pigs (Exp. 1)^a^

Date	CON	R150	R220	R275	SEM^b^	Contrast^c^		
						1	2	3
d 1	3.69	3.68	3.61	3.60	0.04	0.25	0.23	0.64
d 2	3.60	3.63	3.59	3.58	0.04	0.92	0.36	0.79
d 3	3.59	3.55	3.51	3.50	0.03	0.10	0.31	0.77
d 4	3.49	3.46	3.44	3.43	0.03	0.13	0.31	0.84
d 5	3.53	3.50	3.46	3.49	0.02	0.16	0.73	0.32
d 6	3.53	3.49	3.44	3.39	0.03	0.02	0.03	0.98
d 7	3.60	3.58	3.39	3.34	0.03	<0.0001	<0.0001	0.05
Average	3.56	3.55	3.49	3.47	0.03	0.41	0.35	0.14

^a^Abbreviation: *CON* basal diet, *R150* basal diet + nucleotides 150 mg/kg, *R220* basal diet + nucleotides 220 mg/kg, *R275* basal diet + nucleotides 275 mg/kg

^b^Standard error mean

^c^Contrast: 1) CON vs. mean of nucleotide groups; 2) R150 vs. R275; 3) R220 vs. mean of R150 and R275

On d 6, and 7, fecal score in the nucleotide groups was lower (*P* < 0.05) than in the CON treatment and the fecal score in the R275 treatment was lower (*P* < 0.05) than in the R150 treatment. On d 7, the mean fecal score for R150 and R275 treatments was higher (*P* < 0.05) compared with the R220 treatment.

### Exp. 2: Challenge trial

#### Blood profiles, fecal microflora, and fecal score (Tables 7, 8, 9, and 10)

**Table 7:** The effects of dietary nucleotides supplementation on growth performance of weaned pigs (Exp. 2)^a^

Item	CON	R150	R220	R275	SEM^b^	Contrast^c^		
						1	2	3
Body weight, kg								
day 1	8.40	8.40	8.40	8.40	0.01	0.94	0.87	0.74
day 14	13.14	13.27	13.18	13.42	0.11	0.49	0.66	0.51
day 42	23.16	24.10	25.01	25.50	1.21	0.02	0.12	0.27
Before challenge (d 1 to 14)								
ADG, g	339	348	341	359	13	0.07	0.11	0.17
ADFI, g	484	496	491	506	7	0.65	0.42	0.27
G/F	0.700	0.702	0.695	0.709	0.06	0.57	0.43	0.22
After challenge (d 15 to 42)								
ADG, g	357	387	423	431	23	<0.001	0.01	0.33
ADFI, g	566	583	617	626	29	0.03	0.01	0.53
G/F	0.631	0.664	0.686	0.688	0.019	<0.001	0.08	0.77
Overall (d 1 to 42)								
ADG, g	351	374	395	407	20	<0.001	0.01	0.09
ADFI, g	525	540	554	566	11	0.09	0.12	0.60
G/F	0.669	0.693	0.713	0.719	0.016	<0.001	0.06	0.72

^a^Abbreviation: *CON* basal diet, *R150* basal diet + nucleotides 150 mg/kg, *R220* basal diet + nucleotides 220 mg/kg, *R275* basal diet + nucleotides 275 mg/kg

^b^Standard error mean

^c^Contrast: 1) CON vs. mean of nucleotide groups; 2) R150 vs. R275; 3) R220 vs. mean of R150 and R275

**Table 8:** The effects of dietary nucleotides supplementation on blood profiles of weaned pigs challenged with *E. coli K88* (Exp. 2)^a^

Item	CON	R150	R220	R275	SEM^b^	Contrast^c^		
						1	2	3
Before Challenge								
Lymphocyte, %	25.49	30.15	30.92	33.08	1.95	0.01	0.30	0.77
RBC, 10^6^/μL	6.57	6.54	7.00	6.40	0.21	0.75	0.63	0.05
WBC, 10^3^/μL	16.13	17.91	17.73	18.41	0.91	0.08	0.70	0.70
IgG, mg/dL	173.10	184.70	204.10	210.50	15.98	0.16	0.26	0.74
IgA, mg/dL	41.00	45.00	44.40	45.50	1.44	0.02	0.81	0.63
IgM, mg/dL	12.40	14.00	16.70	18.00	1.90	0.09	0.15	0.76
Cortisol	3.47	2.71	2.41	2.22	0.26	0.002	0.19	0.87
TNF-α	116.06	102.82	112.49	124.86	8.36	0.78	0.07	0.90
IGF-I	123.55	148.08	142.95	153.42	11.17	0.07	0.74	0.57
IL-6	19.68	20.49	20.62	19.93	0.79	0.47	0.62	0.68
After Challenge								
Lymphocyte, %	29.30	19.90	29.50	30.89	5.36	0.69	0.16	0.53
RBC, 10^6^/μL	6.33	6.61	6.63	6.54	0.19	0.23	0.79	0.79
WBC, 10^3^/μL	17.33	23.89	18.70	20.92	1.71	0.06	0.23	0.09
IgG, mg/dL	225.50	185.10	220.80	216.40	10.60	0.15	0.05	0.13
IgA, mg/dL	41.90	42.80	44.80	46.20	0.78	0.01	0.004	0.76
IgM, mg/dL	17.30	18.40	24.00	30.00	2.00	0.01	<0.001	0.93
Cortisol	4.17	2.81	3.01	2.72	0.40	0.01	0.87	0.62
TNF-α	173.43	157.33	155.89	148.74	5.82	0.01	0.31	0.69
IGF-I	81.55	113.71	122.27	131.04	7.50	<0.001	0.11	0.99
IL-6	28.23	27.31	25.59	21.51	1.53	0.06	0.01	0.53

^a^Abbreviation: *CON* basal diet, *R150* basal diet + nucleotides 150 mg/kg, *R220* basal diet + nucleotides 220 mg/kg, *R275* basal diet + nucleotides 275 mg/kg

^b^Standard error mean

^c^Contrast: 1) CON vs. mean of nucleotide groups; 2) R150 vs. R275; 3) R220 vs. mean of R150 and R275

**Table 9:** The effects of dietary nucleotides supplementation on fecal microflora of weaned pigs challenged with *E. coli K88* (Exp. 2)^a^

Item, log_10_cfu/g	CON	R150	R220	R275	SEM^b^	Contrast^c^		
						1	2	3
Before Challenge								
*Lactobacillus*	7.23	7.32	7.37	7.44	0.16	0.45	0.60	0.97
*E. coli*	6.14	6.08	6.09	6.07	0.14	0.72	0.98	0.95
After Challenge								
*Lactobacillus*	7.20	7.24	7.27	7.15	0.07	0.77	0.34	0.33
*E. coli*	6.55	6.38	6.34	6.30	0.08	0.03	0.49	0.94

^a^Abbreviation: *CON* basal diet, *R150* basal diet + nucleotides 150 mg/kg, *R220* basal diet + nucleotides 220 mg/kg, *R275* basal diet + nucleotides 275 mg/kg

^b^Standard error mean

^c^Contrast: 1) CON vs. mean of nucleotide groups; 2) R150 vs. R275; 3) R220 vs. mean of R150 and R275

**Table 10:** The effects of dietary nucleotides supplementation on diarrhea score of weaned pigs challenged with *E. coli K88* (Exp. 2)^a^

Date	CON	R150	R220	R275	SEM^b^	Contrast^c^		
						1	2	3
d 21	3.89	3.79	3.75	3.73	0.03	0.0004	0.15	0.78
d 28	3.68	3.58	3.57	3.54	0.03	0.002	0.34	0.78
d 35	3.50	3.42	3.43	3.38	0.03	0.02	0.37	0.43
d 42	3.30	3.13	3.13	3.10	0.03	<0.0001	0.48	0.69
Average	3.60	3.49	3.49	3.44	0.02	<0.0001	0.15	0.40

^a^Abbreviation: *CON* basal diet, *R150* basal diet + nucleotides 150 mg/kg, *R220* basal diet + nucleotides 220 mg/kg, *R275* basal diet + nucleotides 275 mg/kg

^b^Standard error mean

^c^Contrast: 1) CON vs. mean of nucleotide groups; 2) R150 vs. R275; 3) R220 vs. mean of R150 and R275

On d 42, piglets fed the nucleotides supplemented diets had higher (*P* < 0.05) BW compared with those fed the CON diet. Before challenge (from d 1 to 14), no significant difference was observed in growth performance among treatments, whereas, after challenge (from d 15 to 42), ADG, ADFI and G/F ratio of pigs fed nucleotides supplemented diets were higher (*P* < 0.05) compared with those fed the CON diet. Among the nucleotides supplemented groups, ADG and ADFI in the R275 treatment were higher (*P* < 0.05) than in the R150 treatment. Overall (d 1 to 42), ADG and G/F ratio of pigs fed the nucleotides supplemented diets were higher (*P* < 0.05) compared with those fed the CON diet, whereas ADG in the R275 treatment was higher (*P* < 0.05) than that in the R150 treatment.

Before challenge, lymphocyte and IgA levels were higher (*P* < 0.05) in piglets fed the nucleotides supplemented diets compared with CON. Cortisol concentration was lower (*P* < 0.05) in the nucleotide groups compared with the CON group. After challenge, IgA, IgM, and IGF-I concentrations were higher (*P* < 0.05) in the nucleotide groups compared with the CON group. Cortisol and TNF-α concentrations were lower (*P* < 0.05) in the nucleotide groups compared with the CON group. The IgG, IgA and IgM concentrations in R275 treatment were higher (*P* < 0.05) than in the R150 treatments whereas IL-6 levels in the R275 treatment was lower (*P* < 0.05) than in the R150 treatment. No differences (*P* > 0.05) were observed in fecal microflora in challenged pigs among dietary treatments. After challenge, the nucleotides groups had a lower (*P* < 0.05) average fecal score (average of fecal score on d 32, 28, 35, 42) compared with the CON group.

## Discussion

Dietary nucleotides supplementation has been reported to exert positive effects on performance and productivity of pigs. For instance, it was demonstrated that diets supplemented with a nucleotide base at 0.5 % increased ADFI and ADG of weaned pigs [17]. Compared with previous studies, the results of the current study indicate that supplementation of nucleotides in a weaned pig diet improved growth performance and feed efficiency. According to previous studies, nucleotide could stimulate growth and functions of the small intestine [5, 18]. Furthermore, it has also been reported that colostrum or milk nucleotides, as biological active compounds, may influence growth performance and voluntary feed intake of piglets [19–21]. These may be the reasons why dietary nucleotides lead to better growth performance of weaned pigs. However, it was reported that dietary nucleotides supplementation at 0.1 % failed to improve growth performance of weaned pigs [22] and no benefit effects were found when glutamate and nucleotides were used either separately or together in post-weaned piglets [23]. The discrepancy in the findings could possibly be due to the differences in the resources and dosages of nucleotides used in different studies.

During the post-weaning period, gut functions of pigs are very critical in pig production [24]. Diets containing nucleotides maintained a stable microbiota in the ileum [9], which is in line with the finding of the present study showing increased fecal *Lactobacillus* and decreased *E. coli* in the nucleotides treatments. In agreement with our findings, previous study also reported that dietary nucleotides could reduce concentration of enterobacteria and increase number of probiotic bacteria (i.e. *L. acidophilus* and *Bifidobacterium spp.*), which may also result in better intestinal morphology and nutrient uptake [25]. Furthermore, in line with the result of the present, it was reported that a yeast culture product improved the digestibility of DM and CP and the net energy content in piglets [26].

The gastrointestinal tract is the most metabolically active cell mass in animals, with the entire mucosal mass regenerating every 2 to 3 days [27]. However, many factors may cause villous atrophy and increased crypt depth after weaning [28, 29]. The ability of dietary nucleotides supplementation to prevent on villi atrophy in newly weaned pigs has been demonstrated [5]. In pediatric nutrition, it has been demonstrated that nucleotide supplementation reduces the number of episodes of diarrhea [30]. Other studies have shown that dietary nucleotides have beneficial effects on the development of gastrointestinal tract during recovery from diarrhea [11, 31]. Results of these studies implies that dietary sources of nucleotides play an important role in developing, maintaining, and enhancing the immune system, which explains why in the current study fecal score was higher in the CON group compared to nucleotides supplemented groups. It could be argued that reduction of diarrhea could be a direct consequence of better intestinal maturation because of nucleotide supplementation.

Supplementating purified nucleotides to milk replacers for newborn bull calves challenged with lipopolysaccharide (LPS) resulted in calves that tended to have higher mean IgG levels compared to the non-supplemented calves [32]. Pigs with pathogenic *E. coli* infection fed diets supplemented with yeast extract as a source of nucleotides, had reduced diarrhea and improved weight gain and feed conversion ratio compared to pigs fed the control diet [33]. Positive effect of nucleotide supplementation on stimulation of systemic immunity was demonstrated [4]. In the current study after *E. coli* challenge, IgA, IgM, IGF-I were higher and cortisol was lower in pigs fed diets with nucleotide supplementation. In response to challenge, TNF-α, one of the proinflammatory cytokines, decreased in plasma when nucleotides were added in diet. According to previous studies, an appropriate amounts of cytokines, such as IL-1β, IL-6 and TNF-α, is beneficial in response to infection, but overstimulation of the immune system can have detrimental effects. This is because cytokines, especially TNF-α, would cause pathological responses that occur in inflammatory conditions, and therefore it is important to decrease the production of proinflammatory cytokines in order to alleviate inflammation [34]. While cortisol, glucocorticoid which has a potential immune-suppressive and proinflammatory cytokines regulatory effect, also decreased in nucleotide treatments [35]. In line with our result, others have reported that dietary nucleotides could increase plasma immunoglobulins in weaned pigs [22]. This may be due to the fact that nucleotides have been shown to increase T-cell functions and enhance autophagy capacity of pigs [36, 37]. Moreover, nucleotide supplementation led to higher levels of macrophages in the intestine and intra-epithelial lymphocytes [5], which have known to have pathogen-specific cytotoxic T-cell activity [38] and may be able to mediate delayed-type hypersensitivity responses [39]. Therefore, improvement in immunoglobins after an immune challenge may also lead to an improvement in the immune system and better performance of animals fed diets supplemented with nucleotides, which may explain the better growth performance of pigs in the nucleotide groups after an *E. coli* challenge.

## Conclusions

Dietary nucleotides supplementation could help improve growth performance, nutrient digestibility, immune status, microbial balance, reduce diarrhea, and enhance immunity against enterotoxigenic *E. coli* K88 infection in weaning pigs.
